# Implementing successful interprofessional communication opportunities in health care education: a qualitative analysis

**DOI:** 10.5116/ijme.5290.bca6

**Published:** 2013-12-22

**Authors:** Kathryn B. Keller, Terry L. Eggenberger, Julia Belkowitz, Mira Sarsekeyeva, Amalinnette R. Zito

**Affiliations:** 1 Christine E. Lynn College of Nursing at Florida Atlantic University, Boca Raton, Florida, USA; 2 University of Miami Miller School of Medicine, Regional Medical Campus. Boca Raton, Florida, USA; 3 Charles E. Schmidt College of Medicine at Florida Atlantic University. Boca Raton, Florida, USA

**Keywords:** Interprofessional communication, TeamSTEPPS^TM^

## Abstract

**Objectives:**

To explore the experience of an interprofessional communication educational intervention among nursing and medical students.

**Methods:**

Forty-five medical students and 50 nursing students participated in two-hour-long interprofessional communication skills education sessions with interprofessional groups of 6-8 students each. The sessions were based on the Strategies and Tools to Enhance Performance and Patient Safety (TeamSTEPPS^TM^) curriculum. Problematic communication scenarios were presented and then reenacted by the students with role plays that depicted improvements in interprofessional communication. Afterward, narratives describing their experience were collected from a focus group interview. Using the conventional content analysis approach, key phrases and statements were coded into themes.

**Results:**

The study found that students felt increased competence and confidence when responding to conflict after practicing communication in a safe environment. Based on the opportunity to come to know their colleagues, students recognized that patient safety was a shared goal. Six themes were extracted from the narratives describing their experiences: support for process, patient safety, coming to know colleague, support for tools, respectful collaboration, and barriers to communication.

**Conclusions:**

TeamSTEPPS^TM^ provided a framework for effective and respectful collaboration. A significant barrier identified by students was that these communication techniques were not consistently demonstrated during their clinical experiences. An emphasis on interprofessional communication skills and teamwork should begin in the academic setting and be reinforced in both the formal and hidden curricula.

## Introduction

The quality of patient care is improved when members of the health care team work in collaboration to share their unique patient care perspectives.^1^ Each profession enters into practice with different skill sets, knowledge, and professional identities to enhance the care of the patient, yet many barriers exist between disciplines that can obstruct a team-based system.^2^^,^^3^^,^^4^ These barriers include a lack of interprofessional cultural competence, perceived power differentials, and profession-centric role models. In response to these challenges, our faculty found it essential to implement the recently developed Core Competencies for Interprofessional Collaborative Practice in an effort to generate trust, respect, shared accountability and decision-making, and effective teamwork to optimize patient care.^5^ It has been well documented that implementing collaborative interprofessional practices promotes greater patient satisfaction, improved efficiency, and enhanced job satisfaction among healthcare professionals.^6^^,^^7^The Center for Advancement of Interprofessional Education (CAIPE) has defined “interprofessional education (IPE) as occurring when two or more professions learn with, from and about each other to improve collaboration and the quality of care.”^8^ IPE teaches health-care providers to utilize this collaborative approach in clinical settings in order to jointly make decisions, coordinate patient treatment, combine resources, and develop common goals.^9^ The Carnegie Foundation has published two studies that recommend that healthcare educators create new education models that teach their students not only to collaborate with one another, but also to form teams with shared goals to improve patient outcomes.^10^^,^^11^ A team is defined as “a small group of interdependent people who collectively have the expertise, knowledge and skills needed for a task or ongoing work.”^6^ Effective teams must cultivate these critical interprofessional communication behaviors in order to achieve efficient, safe outcomes.^5^^,^^12^^,^^13^ Although this study describes a perspective grounded in the United States health care system, this information is globally relevant since health delivery worldwide has the commonality of interprofessional teams. Curriculum and research concepts from this paper can be replicated to fit within an international medical education framework.The Expert Panel report from the Interprofessional Education Collaborative identified communication as a distinct core competency.^5^ Historically, each health care field has been educated with its own specific language, and this can impede communication when professionals leave the classroom and enter acute-care and community settings. The panel reported that professional jargon can also create a barrier to effective interprofessional care and recommended the use of a common language for team communication, such as the Strategies and Tools to Enhance Performance and Patient Safety (TeamSTEPPS^TM^) curriculum.^14^ Recognizing the importance of these established communication tools, our faculty team developed communication workshops centered around the core aspects of TeamSTEPPS^TM^ and emphasizing the importance of having a shared mental model in our IPE endeavors. The faculty had a mutual interest in learning more about the outcomes of these activities from the students’ perspectives. A study was designed to (a) describe the experience of a joint education intervention between nursing and medical students and (b) explore the students’ perspectives on interprofessional communication and collaboration.

## Method

### Study design

This was a qualitative study utilizing a focus group research design. Focus group methods include interviews in group settings that are led by a facilitator to obtain information about the perceptions, beliefs, ideas, and attitudes of the participants.^15^ Four two-hour-long IPE workshops were conducted, concluding with focus groups facilitated by members of the workshop faculty. The study was approved by the Institutional Review Board at each participating university.

### Description of participants

Participants included 45 medical students and 50 nursing students (see Table 1). The medical students were all in their second year, and the IPE workshop was a part of their required weekly learning community seminar. Rather than participating in their usual communication lab at the medical school, the medical students came to the College of Nursing for one session. The nursing students were senior traditional nursing students and accelerated nursing students in their final semester of study. This experience was not graded, although the nursing students received clinical hours as a part of their leadership practice experience. Neither group had any previous experience with a formalized interprofessional communication program.

**Table 1:** Demographic data

Variable		Medical Students (n=45)	Nursing Students (n=50)
Gender			
	Female	22	39
	Male	23	11
Age (years)			
	20–29	37	38
	30–39	7	9
	40–49	1	3
Previous degrees			
	Bachelor	45	30
	Masters	5	2
	Doctorate	1	0
Previous healthcare experience		19	18

### Data collection process

The nursing and medical students were brought together as part of a communication workshop. An average of 23 students participated in each workshop. The first 30 minutes consisted of a didactic presentation on tools and techniques for interprofessional communication using the TeamSTEPPS^TM^ curriculum. Following the presentation, the students were equally divided into interprofessional groups of 6–8 participants. Each group was provided with a clinical communication scenario that contained many opportunities for improvement. The four clinical scenarios were: 1) anaphylaxis after administration of a medication to which there was a documented allergy, 2) improper CPR administration at a long-term care facility, 3) disrespectful communication between members of a healthcare team in a video clip from a popular television show, and 4) ineffective communication during an early-morning phone call from a nurse to a physician to report a change in patient status. The groups were instructed to use the TeamSTEPPS^TM^ tools to revise and improve the communication outcomes. Additionally, each group was instructed to create a role play depicting the more effective communication methods that they developed for the scenario. These role plays were then presented to the larger group. The original scenarios were presented to the audience just prior to the role plays so that they could see how the tools had been applied to improve communication. A debriefing providing immediate feedback to the participants was conducted after each presentation.When all of the role players had been debriefed, their informed consent for the focus group was obtained, based on a verbal consent script. Students were informed that participation was voluntary, that focus group dialogues would be audio recorded and transcribed with no identifying information, and that all findings would be reported as group data. Demographic data forms were distributed for completion by the students. A semi-structured interview guide was utilized to facilitate dialogue consisting of the following six questions:

1. How do you feel this experience has enhanced your interprofessional communication skills?
2. Can you describe situations that you have seen in practice where you could have utilized the tools you’ve learned today to make a difference?
3. How effective is SBAR [Situation-Background-Assessment-Recommendation and Request; part of the TeamSTEPPS^TM^ curriculum] as a communication tool?
4. Is simulation/role play an effective way to enhance communication skills?
5. How do you think this experience will allow you to contribute to a culture of safety?
6. How has this interaction increased your understanding of the other discipline’s role?

Based upon student feedback at the end of the first session, the didactic session format for the remaining sessions was changed. Students requested information on how each discipline was educated. Faculty from each school explained the educational trajectory of their discipline during the didactic session.

### Data analysis

Three medical and two nursing school faculty analyzed the data using conventional content analysis.^16^ Each faculty member independently read and analyzed the transcribed interviews line by line, highlighting words, key phrases, and statements describing the experience of participating in this interprofessional workshop. The codes that emerged from the initial data analysis are shown in Table 2. Peer review of the data was done in multiple group sessions to verify the consistency of the findings, for confirmability. The discussion in these groups served to increase credibility.^17^ Credibility was also strengthened by a process of reflexivity in which each faculty member reflected on how values, assumptions, and personal beliefs could influence the findings. Additionally, we met as a group to reflect on the data in order to establish evidence of relationships between codes and to confirm and disconfirm themes.^18^ Saturation was reached when the descriptions of the experiences produced no new information. Table 3 indicates the codes that were collapsed into the final themes.

**Table 2:** Codes

Code	Number of times mentioned
Collaboration	10
Respect	11
Team	15
Barriers	21
Support for educational process	25
Safe environment	4
Confidence in clinical	3
Perspective	16
Coming together	16
TeamSTEPPS^TM^ tools	23
Clear communication	11
Patient priority	6
Accountability	3
Safety	6

**Table 3:** Final themes that emerged from codes

Themes	Codes
Support for the process	Safe environment Confidence in clinical Support for educational process
Patient safety	Safety Patient priority Accountability Clear communication
Coming to know colleague	Perspective Coming together
Support for tools	TeamSTEPPS^TM^ tools
Respectful collaboration	Respect Team Collaboration
Barriers to communication	Barriers

## Results

The perspectives of the medical and nursing students after the IPE workshop revealed many descriptive insights. The final six themes that emerged from the data were support for the process, patient safety, coming to know colleague, support for tools, respectful collaboration, and barriers to communication.

### Support for the process

The data revealed that students were highly supportive of this workshop’s format for providing interprofessional education. The codes contributing to this theme were safe environment, confidence in clinical, and support for the educational process. Several students discussed how they felt more comfortable and confident as the result of having an opportunity to practice for what could potentially be emotionally charged situations. One student stated:

“It’s a lot more comfortable doing it in a situation like this first and then when you are in the hospital, you are much more comfortable doing it.” *(Female, nursing student)*

Others elaborated on how the experience allowed them to strive for the ideal communication techniques to use in practice:

“It helps to make the situation seem more real and it … ties you in with the emotions you realize are in practice so that you don’t feel so surprised when it happens in real life … you will be able to expect what is going to happen … when we actually go through the motions of saying the right words and choosing the right phrases and asking the right question … why can’t every conversation be like … this is the ideal—let’s strive to reach it.” *(Female, medical student)*

This participant’s comment reflects on the importance of students recognizing and endorsing the rigor of this exercise for guiding their future practice. There was a consensus that

“… we should definitely train together more; it gives both professions a better understanding of what is going to be expected and how to have better communication with one another.” *(Female, nursing student)*

### Patient safety

Patient priority, safety, accountability, and clear communication were the codes that shaped the theme of patient safety. To achieve effective communication that promotes patient safety, the students felt that they needed to focus on their mutuality of purpose rather than reacting to feelings. The following quotations illustrate the importance of making the patient the priority:

“You need to leave your pride at the door, you need to understand that the patient is the priority and as a doctor being told you made a mistake from a nurse I guess it could have created a tense situation, one, the nurse not wanting to tell, two, the doctor not wanting to hear it. You need to understand that you have the same goal and no one is blaming anyone. Everyone’s here to catch the problem and effectively communicate.” *(Male, medical student)*

“It felt good because it seemed like we were all on the same page, we all understood what the problems were regardless of what our discipline was. The first thing to do was to worry about the patient.” *(Female, nursing student)*

“I think it also reminds all of us that our egos are not what is the most important thing, patient care is.” *(Male, medical student)*

#### Coming to know colleague

The codes perspective and getting to know merged to become the theme coming to know colleague. There were rich descriptions detailing how this exercise allowed the students to gain a perspective about the other discipline’s role, which was necessary for coming to know each other as colleagues. One nursing student stated:

“I think it really helped understand the pressure that is put on the other side, as far as the physicians go and what their expectations are, I think that helped. I thought about if I was in their shoes how frustrating it might be if you relay information to a nurse, you have to take a deep breath, step back and go how do I say this in a constructive way that will motivate this person to want to seek out that information? It was real interesting to see that perspective.” *(Female, nursing student)*

Students articulated a need to understand how the disciplines were educated. A medical student said:

“I think I’d like to know more about nursing school and how it works and how it’s taught to gain a better perspective of what it’s like from that point of view ... I think it would be helpful as a medical student to be told exactly how nursing school works.” *(Male, medical student)*

Students recognized the importance of opportunities to come together as a group:

“Just the fact that we are medical students and nursing students engaging in these exercises together, it gave me an opportunity to meet with the nursing students and it’s something that I hadn’t done before and in our group we learned that nurses do a whole assessment when the patient is admitted to the hospital and we didn’t know that. So it’s important to learn what nurses do … it’s good that we get on the same wavelength.” *(Male, medical student)*

#### Support for tools

Students were highly supportive of the TeamSTEPPS^TM^ tools and techniques. Although they were only queried regarding the use of SBAR, students brought up other tools in discussion, such as the Two-Challenge Rule, Check-Backs, Cross-Monitoring, Debriefing, and the Describe-Express-Suggest-Consequences (DESC) script. One nursing student stated that she liked the concept of a Check-Back to ensure correctness of communication and for clarification. A medical student discussed the use of the Two-Challenge rule as a way to respectfully intervene in order to improve clinical outcomes:

“I thought that by using the ask two times rule, … that actually was powerful because obviously the CPR wasn’t being done correctly so we asked once politely, we asked the second time and you present a pertinent fact as to why you know you’re certain that there is a problem and then cooler heads prevail.” *(Male, medical student)*

The use of SBAR was seen as a valuable way to structure communication:

“I think in instances where there is a lot going on and maybe chaos can get in the way, having tools like SBAR is a clear and concise way to gather your thoughts and communicate effectively.” *(Female, nursing student)*

SBAR was also seen as a way to enhance interprofessional communication:

“It gave us some tools that we didn’t know about before that will help us to connect better and to be able to better communicate between doctors and nurses.” *(Male, medical student)*

### Respectful collaboration

The initial codes respect, team, and collaboration were collapsed to become the theme of respectful collaboration. Students from both disciplines expounded on the importance of engaging in respectful collaborative relationships. The structured activities promoted this level of discourse. As one student remarked:

“It was a lot of mutual respect, I mean just like when we first came here all the medical students were on one side and nurses were on one side but by the time … I mean look at us now. It’s just a nice feeling, you know, that we can communicate with each other.” *(Female, nursing student)*

Additional comments focused on their willingness to build on the strengths that each member brought to the team:

“Our group … didn’t find it hard at all, we kind of played off each other’s suggestions and looked them up and came to a consensus on everything. I think we worked well together.” *(Female, medical student)*

This educational environment and the support of the faculty gave them the freedom to engage in ways of relating that are not always evident in practice:

“I think this setting is nice because you are not in the hospital. There is not a perceived hierarchy between doctors and nurses and we are all kind of on the same level here and it’s more of a team.” *(Female, nursing student)*

### Barriers to communication

There were consistent descriptions of situations that could impede communication. Participants indicated that they find that the practice environment is not interprofessionally culturally competent. They said that hierarchical power struggles impact nurse-physician communication:

“Unfortunately in the hospital I see that it’s really hard for the nurses to really step up and say this is not right because they feel like they are not going to get recognized or you know it is a blame game, … they feel like they are going to get blamed for something and feel stupid.” *(Male, medical student)*

It is clear that the IPE competencies are not present in the environment described. Another medical student reflected:

“I feel like medicine is almost like a monarchy, you know there’s a king on top and then everyone’s a little bit below … I honestly feel that sometimes the nurses get treated unfairly because the physicians … do that and that’s not fair to them. Where I used to work with nurses as a researcher, there were some nurses that I wouldn’t bring an issue to because they were intimidating so … regardless of job title you need to look at yourself from an outside perspective and make sure that you’re behaving towards people in a way that is going to make you and everyone around you able to carry out their jobs as well.” *(Male, medical student)*

Some comments suggested that levels of communication competency may be related to levels of confidence and experience:

“I think … a lot of tension comes from nurses, there’s a lot of tension because they don’t feel comfortable with their doctors. The nurses that are comfortable with their doctors … can get on the phone, can call and talk to them, I need this, this is going on … and they move easier. Where other nurses they just seem to stress … they don’t have the confidence to call the doctor and deal with whatever the situation is.” *(Female, nursing student)*

There was also discussion regarding a lack of understanding among the professions:

“I think sometimes we don’t know each other, we are scared of each other so there are these communication problems.” *(Female, nursing student)*

Students expressed their sincere desire to change existing communication practices:

“I think it’d be great if we could talk to the doctors at the hospitals where we are right now and talk to them about their communication skills. I think it’s great to start while we are all in school, but you know the people that have been in the field for a couple of years they forget what it is like to be a student. They forget what it was like to have an appreciation of the other half of their team. I think they should have to come to the class too.” *(Female, nursing student)*

Another student stated:

“Where I am, there’s a doctor on the floor where all the nurses will just not call him, they wait on calling him for anything because he will just go off on any nurse for any situation.” *(Female, nursing student)*

Other students suggested that they might encounter barriers when attempting to implement the TeamSTEPPS^TM^ tools and techniques in practice:

“For me it seems like … from a medical student’s perspective that going through the ranks, that we always trying to emulate those above us … an attending physician or resident. It’s great that we’re all getting this experience now but, it seems like … residents and the attendings … should be getting this training with the nurses and those that they are working with so that when we are in the hospital or any other type of health care facility that we see them. We see them wash their hands, we see them communicate with—we see them answering the telephone, and we say that is how a doctor acts, that is how a nurse responds to a doctor versus learning it ourselves and then seeing it out in the field and it’s different.” *(Male, medical student)*

These discussions highlight the need to implement this type of IPE program in the practice setting.

## Discussion

Multiple initiatives that require further exploration emerged from the data. One initiative will further expand on the use of role play as an effective method to model communication competencies. The TeamSTEPPS^TM^ tools provide an evidence-based framework to structure communication, which has been supported in the literature.^19^^,^^20^ These tools remove emotional charge and subjectivity from the communication dialogue so that the participants can effectively work together as a team. Providing a safe environment to practice communication has resulted in increased confidence and competence when responding to situations that involve conflict. This supports the recommendations in the Interprofessional Education Collaborative Practice (IPEC) competencies^5^ that healthcare professionals should have the ability to respectfully engage in conflict resolution.It was evident that students felt this interprofessional educational activity provided opportunities to explore how each discipline contributed to their common goal of providing desirable patient outcomes. Having the time to come together to engage in this activity led to the students’ desire to know more about each other and about the other profession. Knowing and understanding that all communication should be directed toward improved patient outcomes enabled them to communicate more effectively to promote patient safety. Coming to know each other as colleagues enhanced respectful collaboration and communication among the team. Such a rich description of coming to know colleagues has not previously been reported in the literature. The students clearly communicated a desire to know each other better as colleagues, and based on this feedback it will be important to include opportunities to explore each others’ roles and responsibilities and to have a shared mental model (SMM) about “making the patient the priority.” SMMs are used to promote communication and increase team effectiveness.^21^^,^^22^ SMMs inform a team’s ability to maximize the role of each team member in contributing to the delivery of care. This allows the team to adapt behaviors based on team performance and the actions of others. If the team acknowledges the importance of using a SMM, the members can successfully strategize team actions and more effectively coordinate care.Student comments also illustrated the potential barriers to introducing interprofessional communication, namely, those that are taught through the hidden curriculum, the norms and values that are formally and informally role-modeled in academia and sustained in practice environments.^23^ Despite our best efforts as educators, strategies that foster communication and teamwork are not always supported when interprofessional learners are placed into clinical environments. Students discussed valuing these new skill sets but being concerned that many practice professionals do not role-model these behaviors, thus reflecting the existence of the hidden curriculum in real-life settings. The power of the hidden curriculum to overshadow the formal objectives taught in academic teaching settings is a factor that must be taken into consideration as faculty attempt to move their initiatives into practice settings. Sustaining the learning outcomes of this initiative will require ongoing development efforts for providers who have not received similar grounding in their educational experience. Faculty must critically evaluate their initiatives and extend these endeavors to the practice setting in order to decrease the gap between academia and practice. A recent review of the concept of the hidden curriculum supports exploring how academic culture and norms shape faculty development.^24^ Interprofessional initiatives are a new frontier within academia that will necessitate faculty to reflect on their own acculturation to the faculty role and to understand the cultural mores of other disciplines.There are several potential limitations to the methodology of this study. The focus groups were facilitated by the faculty members coordinating and implementing the educational session. These facilitators were not the students’ clinical instructors and were not involved in any type of grading activities. Some students were less participatory than others, which could reflect apprehension or a lack of trust concerning the process. Any limitation stemming from the use of faculty facilitators could be addressed by using outside moderators. The varying levels of participation might also be attributed to the size of the focus groups, since the recommended number of participants varies from a minimum of four to a maximum of twenty,^25^ although instances are cited in the literature where focus group numbers numbered in the twenties.^26^ Because this study’s activity was structured around clinical groups, our workshops had an average of 23 participants. Although we recognize that this is a large number, the majority of students actively participated in the discussions, and this resulted in a rich description of the experience. A recommendation for the future would include dividing the students into smaller groups and running several focus groups concurrently. Self-censoring by students could also reflect variations in clinical experience between the two groups. The nursing students had more hospital-based clinical experience, while the majority of the second-year medical students’ experience had been in the outpatient setting. However, the workshops emphasized broad communication concepts rather than complex clinical knowledge.

## Conclusion

Participants in the study provided rich descriptions of nursing and medical students’ experiences in this interprofessional program. The student’s perspectives included a desire to know more about how each role contributes to patient outcomes. The TeamSTEPPS^TM^ tools were seen as effective techniques for addressing conflict and entering into a respectful collaborative dialogue. Students felt that spending more time together in similar structured activities would increase their confidence about responding to comparable situations in practice.An emphasis on interprofessional competence within the IPEC domains of communication and teamwork must begin in the academic setting and be reinforced/role-modeled throughout the curriculum. This study has stimulated the faculty to begin planning ways to sustain this initiative and to assess how to transmit these values into clinical practice. As a result of working together to plan, implement, and study this communication intervention, we have become cognizant of the importance of spending more time in faculty development. The process begins with interprofessional faculty coming to respect and know one another as colleagues. Then together we can strategize ways for clinical faculty to effectively construct opportunities for students and other clinicians that support IPEC competencies in practice. If these values are formalized and visible, interprofessional competencies will become sustainable curricular outcomes.

## 

### Conflict of Interest

The authors declare that they have no conflicts of interest.
